# Association Between Dental Caries Prevalence and Body Mass Index in Children with and Without Special Needs: A Comparative Study in Jeddah, Saudi Arabia

**DOI:** 10.3390/jcm14124165

**Published:** 2025-06-12

**Authors:** Sakeenabi Basha, Mohammed Khalil Fahmi, Roshan Noor Mohamed, Alaa Redwan, Arwa U. Alsaggaf, Yasser Eid Al Thobaiti, Ali Alqarni, Azzah O. Alhazmi, Yousef Al Thomali, Turky Alayyafi, Khalid A. Bagadeem

**Affiliations:** 1 Department of Preventive Dentistry, Faculty of Dentistry, Taif University, Taif 21944, Saudi Arabia; roshan@tu.edu.sa (R.N.M.); az.alhazmi@tu.edu.sa (A.O.A.); yalthomali@tu.edu.sa (Y.A.T.); 2 Department of Restorative Dental Sciences, Faculty of Dentistry, Taif University, Taif 21944, Saudi Arabia; m.fahmi@tu.edu.sa; 3 Pediatric Dentistry Division, Preventive Dentistry Department, Faculty of Dental Medicine, Umm Al-Qura University, Makkah 24382, Saudi Arabia; akredwan@uqu.edu.sa; 4 Prosthodontic Division, Oral and Maxillofacial Surgery Department, Faculty of Dental Medicine, Umm Al-Qura University, Makkah 24382, Saudi Arabia; ausaggaf@uqu.edu.sa; 5 Department of Oral and Maxillofacial Surgery and Diagnostic Sciences, Faculty of Dentistry, Taif University, Taif 21944, Saudi Arabia; y.thobaiti@tu.edu.sa (Y.E.A.T.); aqarni@tu.edu.sa (A.A.); 6 Faculty of Dental Medicine, Umm Al-Qura University, Makkah 24382, Saudi Arabia; turky.alayyafi@gmail.com; 7 Advanced Restorative Dentistry, Ministry of Health, King Fahd General Hospital Dental Center, Jeddah 23311, Saudi Arabia; dr.bagadeem@hotmail.com

**Keywords:** dental caries, children with disabilities, comparative study, body mass index, obesity

## Abstract

**Objectives:** The present study aims to compare the prevalence of dental caries between children with special needs (CSN) and children without special health care needs (CWSCN), and additionally, this study explores the association between body mass index (BMI) and dental caries in both groups. **Methods:** A cross-sectional descriptive study was conducted. A total of 773 children were selected using the two-stage random sampling method (257 CSN and 516 CWSCN). The World Health Organization criteria was used to diagnose dental caries. BMI was determined by using height and weight measurements. Multivariable logistic regression was used to determine the relationships between dental caries prevalence (yes/no) and independent variables. **Result:** Special needs children had a 2.87 (95% CI: 1.56–4.03, *p* = 0.001) times higher risk of caries compared with CWSCN. Female children had a 1.76 (95% CI: 1.52–3.24, *p* = 0.024) times higher risk of caries than male children. Children who consume sugar frequently were 2.03 (95% CI: 1.73–4.08, *p* = 0.001) times more likely to have caries. Children with obesity were 2.15 (95% CI: 1.81–4.79, *p* = 0.001) times more likely to have caries compared with normal-weight children. Children who used non-fluoridated toothpaste had a 1.92 times (95% CI: 1.68–4.19, *p* = 0.031) higher risk of caries compared with children who used fluoridated toothpaste. **Conclusions:** The present study highlights the higher prevalence of dental caries among CSN compared with CWSCN and identifies several significant risk factors, including gender, parental education, sugar consumption, obesity, and the use of non-fluoridated toothpaste.

## 1. Introduction

Dental caries remains a significant public health challenge, particularly among children, with prevalence rates of 60–90% globally [1]. In Saudi Arabia, recent studies report an average prevalence of 75.43% in primary dentition and 67.7% in permanent dentition among children [2]. The high burden of dental caries in this population underscores the need for targeted interventions, especially for vulnerable groups such as children with special needs (CSN).

Children with special needs (CSN) comprise children living with chronic behavioral, cognitive, physical, and communication difficulties [3]. Globally, more than 1.3 billion people (16% of the global population) are recorded as having special needs status; out of these, 93 million children present with moderate to severe special needs status [4]. In Saudi Arabia, approximately 6.3% of children under 16 years of age are recorded as having a disability [5]. Disparities in oral health outcomes for CSN are well-documented, with higher caries prevalence attributed to barriers in accessing care, inadequate oral hygiene practices, and dietary challenges [6,7,8,9,10,11,12,13]. Local studies, such as those by Alamri H [6], Asiri F et al. [10], and Fahmi et al. [11], highlight the urgent need for tailored oral health strategies for this group.

The etiology of dental caries is multifactorial, involving poor oral hygiene, frequent sugar consumption, inadequate fluoride exposure, and alterations in oral microbiota (e.g., *Streptococcus* spp., *Lactobacillus* spp., *Candida albicans*) [14,15]. Additionally, systemic conditions and medications, such as corticosteroids, can exacerbate caries risk by reducing salivary flow and altering immune responses, as noted by Vitale M. C et al. [16]. Other risk factors include enamel defects, low socioeconomic status, and obesity, the latter of which has been increasingly linked to caries in Saudi children [17,18,19,20].

The relationship between BMI and dental caries is complex and context-dependent. While global evidence is mixed [21,22,23], studies in Saudi Arabia [19,20,24,25,26], such as those by Alshehri et al. [24] and Ashour et al. [27], report a positive association between obesity and caries, particularly among CSN. This population faces unique challenges, including limited physical activity and dietary restrictions, which may further elevate their risks for both obesity and caries [24,27].

The present study aims to compare dental caries prevalence between CSN and children without special needs (CWSCN) in Jeddah, Saudi Arabia, while examining the association between BMI and caries in both groups. By focusing on local data and contextualizing risk factors, the present study seeks to inform targeted interventions for reducing oral health disparities in this region.

The present research is guided by three research questions: A. What is the prevalence of dental caries among children with and without special health care needs? B. What is the relationship between dental caries and body mass index (BMI) in children with special needs compared with children without special needs? C. How do various sociodemographic, dietary, and oral hygiene factors influence the prevalence of dental caries?

## 2. Materials and Methods

Study design and participants: This research employed a cross-sectional descriptive design to evaluate children with and without special needs (CWSCN) in Jeddah, Saudi Arabia, between January and December 2024. A total of 773 participants were enrolled, including 257 children with special needs and 516 children without special needs. The required sample size was calculated using preliminary data from a pilot study involving 20 children with special needs and 30 without special needs. Notably, participants from the pilot phase were excluded from the final study cohort. The sample size of 700 was calculated with a precision of 35% and an error of 5%. To account for a potential non-response bias, the target sample size was adjusted to 750 participants.

Sample methods: This study utilized a two-phase randomized sampling technique for participant selection. In the first stage, 30 schools (6 per region) were randomly selected from five administrative regions of Jeddah (North, South, Central, East, West) using the lottery method. These regions were chosen to reflect Jeddah’s socioeconomic and demographic diversity. In the second stage, children were proportionally sampled from each school (4–12 CSN and 8–24 CWSCN per school) and stratified by age (6–11 and 12–16 years) and special needs status.

Ethical clearance and informed consent: This study received approval from the Institutional Review Board (approval number: HAO-02-T-105) prior to commencement. Informed written consent was secured from the parents or legal guardians of all participants. Additionally, verbal or written assent was acquired from the participating children, as appropriate for their ages and cognitive abilities.

Eligibility criteria: Inclusion criteria were as follows: children aged 6–16 years and the presence of a signed informed consent from a parent or legal guardian. Exclusion criteria were as follows: absence of a written informed consent from a parent/guardian, documented aggressive behavior that could compromise safety, and demonstrated inability to cooperate with dental examination procedures.

Sociodemographic, diet, and oral hygiene information: Based on the previously published literature [23,24,25,26,27], a questionnaire was developed to collect sociodemographic factors (gender, age, parents education, parents occupation, family income), dietary habits (72-h recall data that spanned a weekend and 2 weekdays, for which the number of meals and the form, frequency, consistency, and time of sugar intake were recorded), oral hygiene practices (method of tooth cleaning, material used, frequency of cleaning, use of fluoridated tooth paste), medication history (including xerostomia-inducing drugs), previous dental visits (as a proxy for access to care), and type and duration of disability. The questionnaire was pretested (Cronbach’s alpha α = 0.85).

BMI measurement: BMI was calculated as weight (kg)/height (m^2^) and categorized using the WHO Growth Standards for children aged 5–19 years: Underweight (<−2 SD), Normal (−2 to +1 SD), Overweight (>+1 SD, equivalent to BMI 25 kg/m^2^), Obese (>+2 SD, equivalent to BMI 30 kg/m^2^) [28].

Oral examination: A single calibrated examiner performed all oral assessments under natural lighting conditions, utilizing sterilized plane mouth mirrors and Community Periodontal Index (CPI) probes. Dental caries was evaluated according to the WHO standards [29], with documentation of decayed, missing, and filled teeth/surfaces (dmft/DMFT and dmfs/DMFS indices). While this approach is standardized for epidemiological studies, it may underestimate interproximal caries without radiographic or transillumination support. To mitigate potential misclassification bias, the examiner was rigorously trained and calibrated (kappa = 0.90 for intra-examiner reliability). 

Statistical analysis: The analysis employed a comprehensive analytical framework to examine both proportional and continuous data. For categorical variables, group differences were assessed using Chi-square (χ^2^) tests for nominal data and the nonparametric Kruskal–Wallis H test for ordinal comparisons, with post hoc Mann–Whitney U tests to evaluate subgroup disparities. Continuous variables were analyzed via independent Student’s *t*-tests for two-group comparisons and one-way ANOVA for multi-group means, supplemented by Tukey’s honestly significant difference (HSD) post hoc tests to control for the Type I error. To identify predictors of dental caries prevalence (binary outcome: yes/no), a multivariate logistic regression model was constructed, adjusting for multicollinearity among covariates. The model incorporated demographic predictors (age, gender, special needs status), socioeconomic factors (parental education), behavioral variables (frequency of sugar consumption, toothbrushing habits, fluoride toothpaste use), and physiological measures (BMI categories). All analyses were executed in IBM SPSS Statistics (Version 22; Armonk, NY, USA), with statistical significance defined as two-tailed *p* < 0.05.

## 3. Results

The study cohort comprised 773 participants, including 257 children with special needs and 516 children without special needs (CWSCN). The average age across all participants was 10.8 years (SD = 5.2). Table 1 presents the BMI categories among special needs and CWSCN according to the variables studied. Thirty-one (25.8%) special needs children and 45 (16.4%) CWSCN of age 12–16 years, respectively, presented with obesity. Out of 257 special needs children, 23 (17.4%) boys and 35 (28%) girls presented with obesity. Out of 168 special needs children who consumed sugar frequently, 45 (26.8%) children presented with obesity.

Table 2 shows the mean caries scores among the study participants according to age. The overall mean dmft score was 3.9 ± 2.6, and the dmfs score was 6.0 ± 4.1. The overall DMFT score was 3.9 ± 2.7, and the DMFS score was 4.6 ± 2.7. The mean dmft scores were 4.1 ± 2.8 and 3.6 ± 2.3, respectively, among 6–11-year-old CSN and CWSCN (*p* = 0.04). The mean DMFT scores were 4.2 ± 2.7 and 3.1 ± 1.7, respectively, among 12–16-year-old CSN and CWSCN (*p* = 0.001).

The regression analysis results are presented in Table 3. Special-needs children had a 2.87 (95% CI: 1.56–4.03, *p* = 0.001) times higher risk of caries compared with CWSCN. Female children had a 1.76 (95% CI: 1.52–3.24, *p* = 0.024) times higher risk of caries than male children. Children with parents who had primary school or less education had a 1.78 (95% CI: 1.53–3.87, *p* = 0.041) times higher risk of caries than children with parents who had intermediate or higher education. Children who consumed sugar frequently were 2.03 (95% CI: 1.73–4.08, *p* = 0.001) times more likely to have caries than children who did not consume sugar frequently. Children with obesity were 2.15 (95% CI: 1.81–4.79, *p* = 0.001) times more likely to have caries than normal-weight children. Children who used non-fluoridated toothpaste had a 1.92 times (95% CI: 1.68–4.19, *p* = 0.031) higher risk of caries compared with children who used fluoridated toothpaste.

## 4. Discussion

The present study aimed to compare the prevalence of dental caries between children with special needs (CSN) and CWSCN in Jeddah, Saudi Arabia, and to explore the association between dental caries and body mass index (BMI) in both groups.

Obesity and special needs status: The current study results indicate that 25.8% of CSN and 16.4% of CWSCN aged 12–16 years were obese (Table 1). This higher prevalence of obesity among CSN aligns with previous research suggesting that children with special needs are at greater risk for obesity due to factors such as limited physical activity and dietary challenges [30,31,32,33]. The gender disparity observed, with 28% of girls and 17.4% of boys among CSN being obese, is consistent with studies indicating that girls with disabilities may have higher obesity rates due to hormonal and metabolic differences [33].

Dental caries and special needs status: The mean dmft and DMFT scores were significantly higher among CSN as compared with CWSCN, indicating a greater burden of dental caries in this population (Table 2). Regression analysis revealed that CSN were 2.87 times more likely to develop caries than CWSCN (*p* = 0.0001) (Table 3). This finding is supported by previous studies that have documented higher caries prevalence among children with disabilities, which is attributed to difficulties in maintaining oral hygiene and accessing dental care [34,35,36]. For instance, Tounsi, A et al. [36] reported that CSN in Riyadh, Saudi Arabia, had a higher prevalence of dental caries compared with CWSCN in the same region, which aligns with our findings.

Dental caries and sociodemographic status: In the present study, age, gender, and parental education played significant roles in dental caries prevalence. The female children were 1.76 times more likely to have dental caries compared with male children (*p* = 0.024) [Table 3]. This finding is consistent with some previous studies that have reported a higher prevalence of dental caries among females, possibly due to the earlier eruption of permanent teeth and dietary differences [25,26,27]. However, other studies have found no significant gender differences in caries prevalence, indicating potential heterogeneity in results across different populations and settings [20,23]. The level of education attained by parents was also a key factor linked to the prevalence of dental caries in children. Children whose parents had primary school education or less were 1.78 times more likely to have dental caries compared with those with parents who had intermediate or higher education (*p* = 0.041) (Table 3). This finding is supported by previous research that has identified socioeconomic status, including parental education, as a key determinant of oral health outcomes in children [27,36]. Lower parental education may be associated with poorer oral health knowledge and practices, leading to higher caries prevalence [36].

Sugar consumption and dental caries: Dietary habits, particularly frequent sugar consumption, were strongly associated with dental caries in this study. The present study could not show a direct association between sugar consumption and BMI in either CSN or CWSCN (*p* > 0.05) (Table 1). The lack of a direct association between sugar consumption and BMI in the present study population may reflect the fact that, while sugar intake remains a well-established caries risk factor [21,22], its relationship with obesity appears mediated by broader dietary patterns and physical activity levels, particularly in special needs populations [22]. This finding highlights the importance of comprehensive nutritional assessments beyond simple sugar quantification when evaluating health risks in CSN [21]. Children who consumed sugar frequently were 2.03 times more likely to have dental caries as compared with those who did not (*p* = 0.001). This is consistent with numerous studies that have identified sugar consumption as a major risk factor for dental caries [21,22,27]. The role of sugar in caries development is well-established, and our findings further emphasize the need for dietary interventions to reduce sugar intake among children.

BMI and dental caries: Regarding BMI, this study found that 25.8% of CSN aged 12–16 years were obese, compared with 16.4% of CWSCN in the same age group. In line with previous studies [25,26,27,37], the present study showed a strong association between obesity and dental caries, with obese children having a 2.15 times higher risk of caries compared with normal-weight children. This could be attributed to a shared risk factor—high sugar intake—which may elevate the risk of dental caries in children with obesity. In contrast to this, a few studies have found no significant relationship [20,23]. The heterogeneity in these findings may be due to differences in study populations, methodologies, and the complex interplay between diet, oral hygiene, and BMI. For example, Chen et al. [21] conducted a systematic review and meta-analysis and found a weak but significant association between dental caries and BMI in children, but noted substantial heterogeneity across the findings. A similar report was also substantiated in another systematic review by Alshehri et al. [24], where two of the included studies showed position association, but six studies showed negative association between BMI and dental caries. While the present study demonstrates a significant association between obesity and dental caries, the cross-sectional design precludes definitive causal conclusions due to abovementioned heterogenicity and possible bidirectional relationships.

Oral hygiene practice and dental caries: In the present study, the use of non-fluoridated toothpaste was associated with a higher risk of dental caries, with children who used non-fluoridated toothpaste being 1.92 times more likely to have caries compared with those who used fluoridated toothpaste (*p* = 0.031) (Table 3). This finding aligns with the well-documented protective effect of fluoride against dental caries [38,39,40]. The use of fluoridated toothpaste is a simple yet effective measure for caries prevention, and our results underscore the importance of promoting its use, especially among vulnerable populations like CSN. However, the significant shortcoming of the use of fluoride is the overcoming of safety doses, toxicity, and risk of fluorosis, especially among children [41]. Emerging evidence supports biomimetic hydroxyapatite (HAP) as a safe and effective alternative, particularly for pediatric populations [42]. Recent clinical trials have demonstrated its efficacy in caries prevention, even in children with special needs [42,43].

Consistent with earlier research [44,45], this study found that children who brushed their teeth once a day or less had a higher likelihood of developing dental caries, with an odds ratio of 1.95. This highlights the importance of proper oral hygiene instruction with the use of fluoridated dentifrice for children with and without special needs to reduce the risk of dental caries.

Medication use and dental visits: While our study did not find statistically significant associations between medication use/dental visits and caries in CSN, these factors remain clinically relevant. For medications, the lack of significance may reflect heterogeneous drug effects (e.g., sugar-free formulations or compensatory oral hygiene in medicated children) or insufficient power to detect subgroup differences (e.g., xerostomia-inducing drugs) [9,46]. Similarly, dental visit frequency may not correlate with caries if visits are episodic (emergency-driven) rather than preventive, or if access barriers limit care quality [36,37]. Future studies should stratify medications by cariogenic risk and differentiate visit types (preventive vs. restorative) to clarify these relationships.

Study limitations: This study employed a robust methodology, including a large sample size and a two-stage random sampling technique, which enhances the generalizability of the results. However, the cross-sectional design precludes definitive conclusions about causality. The reliance on visual–tactile methods without radiographs or transillumination may have led to an underestimation of interproximal caries. Future studies could incorporate adjunct diagnostic tools to improve accuracy. Some residual confounding may persist related to medications (e.g., antiepileptics, antihistamines) and disparities in dental care access due to logistical or financial barriers, which may exacerbate oral health inequalities. Future studies should explore these factors in greater depth. Future longitudinal research is necessary to clarify the temporal relationships between dental caries, BMI, and other contributing factors.

## 5. Conclusions

The present study highlights the higher prevalence of dental caries among CSN compared with CWSCN and identifies several significant risk factors, including obesity, gender, parental education, sugar consumption, and the use of non-fluoridated toothpaste. Our findings reveal urgent opportunities to transform oral health care for CSN through the following targeted, collaborative strategies: clinical action through biannual caries risk assessments in pediatric practices, incorporating BMI screening and dietary counseling; prevention programs through school-based initiatives featuring hands-on brushing workshops, fluoride varnish applications, and sugar-reduction education tailored for CSN and caregivers; policy reforms ensuring free, accessible preventive dental care, including mobile clinics for underserved areas, government-subsidized fluoride/hydroxyapatite toothpaste distribution through schools and clinics, mandated biannual dental visits, and national public health campaigns promoting evidence-based caries prevention; and, research priorities focused on comparative studies of preventive agents and cost–benefit analyses of early interventions. This multipronged approach—bridging clinical practice, public health, and policy—can effectively reduce oral health disparities when implemented through cross-sector collaboration.

## Figures and Tables

**Table 1:** Body mass index (BMI) categories among special needs and CWSCN according to the variables studied.

Variable	BMI Categories	CSN (n = 257)	CWSCN (n = 516)	Between-Group *p*-Value (Chi-Square)
6–11 years	Underweight	5 (3.6)	4 (1.7)	0.1272
	Normal weight	83 (60.6)	169 (70.1)	0.082
	Overweight	22 (16.1)	30 (12.4)	0.131
	Obese	27 (19.7)	38 (15.8)	0.092
12–16 years	Underweight	4 (3.3)	7 (2.5)	0.172
	Normal weight	60 (50)	182 (66.2)	0.073
	Overweight	25 (20.8)	41 (14.9)	0.082
	Obese	31 (25.8)	45 (16.4)	0.075
Boys	Underweight	6 (4.5)	8 (2.9)	0.142
	Normal weight	76 (57.6)	181 (67.0)	0.136
	Overweight	27 (20.4)	39 (14.4)	0.112
	Obese	23 (17.4)	42 (15.6)	0.062
Girls	Underweight	3 (2.4)	3 (1.2)	0.172
	Normal weight	67 (53.6)	170 (69.1)	0.091
	Overweight	20 (16)	32 (13.0)	0.112
	Obese	35 (28)	41 (16.7)	0.118
Frequent	Underweight	6 (3.6)	7 (2.8)	0.141
	Normal weight	85 (50.6)	165 (65.2)	0.052
	Overweight	32 (19.0)	37 (14.6)	0.091
	Obese	45 (26.8)	44 (17.4)	0.074
Not frequent	Underweight	3 (3.4)	4 (1.5)	0.113
	Normal weight	58 (65.2)	186 (70.7)	0.082
	Overweight	15 (16.9)	34 (12.9)	0.114
	Obese	13 (14.6)	39 (14.8)	0.091

CSN—children with special needs, CWSCN—children without special needs.

**Table 2:** Mean (standard deviation) caries scores among study participants according to age.

Variables	n	dmft	dmfs	DMFT	DMFS
**CSN**					
6–11-years	137	4.1 (2.8)	6.1 (4.2)	1.5 (0.8)	2.3 (1.5)
CWSCN					
6–11-years	241	3.6 (2.3)	5.4 (3.8)	1.2 (0.6)	1.8 (1.1)
*t*-test, *p*-value		0.04	0.03	0.06	0.04
CSN					
12–16-years	120	0.05 (0.01)	0.08 (0.03)	4.2 (2.7)	5.5 (3.1)
CWSCN					
12–16-years	275	0.01 (0.01)	0.03 (0.01)	3.1 (1.7)	4.4 (2.6)
*t*-test, *p*-value		0.09	0.09	0.001	0.02

Values in parenthesis show standard deviation. CSN—children with special needs, CWSCN—children without special health care needs.

**Table 3:** Multinomial logistic regression analysis with dental caries as a dependent variable.

Variables Studied	Adjusted OR	Lower Bound	Upper Bound	*p* Value	VIF
**Type of study participants**					
CSN	2.87	1.56	4.03	0.001	1.4
CWSCN #					
**Age in years**					
6–11-year #					
12–16-year	1.53	1.41	2.97	0.041	1.2
**Gender**					
Male #					
Female	1.76	1.52	3.24	0.024	1.1
**Parental education**					
Primary school or less education	1.78	1.53	3.87	0.041	1.3
Intermediate or higher #					
**BMI**					
Underweight	0.96	0.72	1.51	0.062	
Normal weight					
Overweight	1.51	1.42	2.89	0.041	1.4
Obese	2.15	1.81	4.79	0.001	1.5
**Sugar consumption**					
Frequent sugar consumption	2.13	1.73	4.08	0.001	1.6
No frequent sugar consumption #					
**Tooth brushing frequency**					
≥two times daily #					
≤once daily	1.95	1.62	4.14	0.032	1.7
**Type of toohpaste used**					
Fluoridated toothpaste #					
Non-fluoridated toothpaste	1.92	1.68	4.19	0.031	1.3
Don’t know	0.75	0.32	1.02	0.072	
**Visit to dentist**					
Yes #					
No	0.82	0.53	1.21	0.063	1.2
**Use of medications**					
Yes	0.93	0.61	1.29	0.061	1.1
No #					

#—Reference, OR: odds ratio, CSN—children with special needs, CWSCN—children without special needs, BMI—body mass index, VIF—variance inflation factor, Hosmer–Lemeshow Test, *p* = 0.41.

## Data Availability

The original contributions presented in this study are included in the article. Further inquiries can be directed to the corresponding author.

## Abbreviations

CSN	Children with special needs
CWSCN	Children without special needs
